# Supplementary material for: Familial Risks and Proportions Describing Population Landscape of Familial Cancer
Source: Cancers (Basel). 2021 Aug 30;13(17):4385. doi: 10.3390/cancers13174385 (PMC8430802; doi:10.3390/cancers13174385)
Supplement: Supplementary file 1 [file cancers-13-04385-s001.zip › cancers-1296917-supplementary.pdf]

Supplementary tables:

Supplementary Table 1. Age-specific incidence rate and rate ratio of stomach and colorectal cancer in population with and without family history of concordant cancer

Supplementary Table 2. Age-specific incidence rate and rate ratio of pancreas and lung cancer in population with and without family history of concordant cancer

Supplementary Table. Age-specific incidence rate and rate ratio of female breast and endometrium cancer in population with and without family history of concordant cancer

Supplementary Table. Age-specific incidence rate and rate ratio of prostate and kidney (Renal parenchyma) cancer in population with and without family history of concordant cancer

Supplementary Table. Age-specific incidence rate and rate ratio of bladder (Urinary bladder) cancer and melanoma in population with and without family history of concordant cancer

Supplementary Table 1. Age-specific incidence rate and rate ratio of stomach and colorectal cancer in population with and without family history of concordant cancer

	Stomach					Colorectum				
	Incidence rate per 100 000 person years		Rate ratio (Familial/Non-familial)			Incidence rate per 100 000 person years		Rate ratio (Familial/Non-familial)		
Age at diagnosis (years)	Familial	Non-familial	RR	95% CI		Familial	Non-familial	RR	95% CI	
20-24	0.4	0.1	4.2	1.0	17.4	1.4	1.2	1.2	0.8	1.7
25-29	1.2	0.2	5.2	2.2	12.0	2.9	1.8	1.6	1.2	2.1
30-34	0.4	0.4	0.9	0.2	3.8	5.2	2.5	2.1	1.7	2.6
35-39	1.7	0.8	2.1	1.0	4.3	9.2	4.4	2.1	1.8	2.5
40-44	3.2	1.6	2.0	1.2	3.3	19.1	8.3	2.3	2.0	2.6
45-49	7.7	3.0	2.5	1.8	3.6	29.5	14.8	2.0	1.8	2.2
50-54	10.0	5.1	2.0	1.4	2.7	55.6	28.4	2.0	1.8	2.1
55-59	17.1	9.0	1.9	1.5	2.4	91.8	51.4	1.8	1.7	1.9
60-64	26.2	12.7	2.1	1.6	2.6	146.9	87.9	1.7	1.6	1.8
65-69	35.7	18.3	1.9	1.6	2.4	213.7	134.5	1.6	1.5	1.7
70-74	31.6	24.1	1.3	1.0	1.8	315.9	192.1	1.6	1.5	1.8
75-79	48.3	26.8	1.8	1.2	2.6	434.8	268.2	1.6	1.5	1.8
80-84	44.7	33.6	1.3	0.6	3.0	427.9	338.0	1.3	1.1	1.5

RR: Rate ratio; CI: confidence interval.

Supplementary Table 2. Age-specific incidence rate and rate ratio of pancreas and lung cancer in population with and without family history of concordant cancer

	Pancreas					Lung				
	Incidence rate per 100 000 person years		Rate ratio (Familial/Non-familial)			Incidence rate per 100 000 person years		Rate ratio (Familial/Non-familial)		
Age at diagnosis (years)	Familial	Non-familial	RR	95% CI		Familial	Non-familial	RR	95% CI	
20-24	0.2	0.1	4.5	0.6	34.1	0.2	0.2	1.1	0.4	3.7
25-29	0.7	0.1	9.2	2.7	30.8	0.8	0.4	2.1	1.1	4.0
30-34	1.0	0.2	6.1	2.2	16.9	0.7	0.6	1.2	0.6	2.4
35-39	0.2	0.4	0.6	0.1	4.1	2.6	1.2	2.1	1.4	3.0
40-44	2.9	1.1	2.7	1.5	5.0	7.7	3.0	2.6	2.1	3.3
45-49	6.9	2.5	2.8	1.8	4.1	19.3	8.1	2.4	2.1	2.8
50-54	14.6	6.0	2.5	1.8	3.3	47.2	18.6	2.5	2.3	2.8
55-59	29.8	12.0	2.5	2.0	3.1	84.0	38.9	2.2	2.0	2.3
60-64	40.5	19.7	2.1	1.7	2.5	154.6	68.6	2.3	2.1	2.4
65-69	53.5	29.8	1.8	1.4	2.2	239.4	102.9	2.3	2.2	2.5
70-74	73.4	41.6	1.8	1.4	2.2	287.3	138.2	2.1	1.9	2.3
75-79	67.3	50.5	1.3	0.9	1.9	306.3	154.7	2.0	1.7	2.2
80-84	187.8	57.4	3.3	2.0	5.5	286.8	167.8	1.7	1.3	2.3

RR: Rate ratio; CI: confidence interval.

Supplementary Table 3. Age-specific incidence rate and rate ratio of breast (women) and endometrium cancer in population with and without family history of concordant cancer

	Breast (women)					Endometrium				
	Incidence rate per 100 000 person years		Rate ratio (Familial/Non-familial)			Incidence rate per 100 000 person years		Rate ratio (Familial/Non-familial)		
Age at diagnosis (years)	Familial	Non-familial	RR	95% CI		Familial	Non-familial	RR	95% CI	
20-24	1.3	0.5	2.5	1.4	4.3		0.1			
25-29	9.7	3.7	2.6	2.1	3.2	0.8	0.3	2.9	0.7	12.2
30-34	30.7	14.5	2.1	1.9	2.4	1.6	0.8	1.9	0.7	5.3
35-39	69.1	36.3	1.9	1.8	2.1	4.5	2.3	2.0	1.1	3.6
40-44	162.2	80.7	2.0	1.9	2.1	15.3	4.8	3.2	2.2	4.4
45-49	283.6	152.4	1.9	1.8	1.9	36.6	12.4	2.9	2.3	3.7
50-54	359.5	203.9	1.8	1.7	1.8	59.0	27.6	2.1	1.8	2.6
55-59	413.2	239.7	1.7	1.7	1.8	95.9	50.8	1.9	1.6	2.2
60-64	506.4	303.6	1.7	1.6	1.7	134.7	66.0	2.0	1.7	2.4
65-69	599.3	356.6	1.7	1.6	1.8	133.8	87.1	1.5	1.3	1.8
70-74	614.9	382.6	1.6	1.5	1.7	159.1	98.4	1.6	1.3	2.0
75-79	453.8	257.9	1.8	1.6	2.0	181.4	111.2	1.6	1.2	2.3
80-84	442.3	343.3	1.3	1.0	1.7	193.1	137.1	1.4	0.7	2.8

RR: Rate ratio; CI: confidence interval.

Supplementary Table 4. Age-specific incidence rate and rate ratio of prostate and kidney cancer in population with and without family history of concordant cancer

	Prostate					Kidney				
	Incidence rate per 100 000 person years		Rate ratio (Familial/Non-familial)			Incidence rate per 100 000 person years		Rate ratio (Familial/Non-familial)		
Age at diagnosis (years)	Familial	Non-familial	RR	95% CI		Familial	Non-familial	RR	95% CI	
20-24		0.0				0.3	0.1	2.7	0.4	20.1
25-29		0.0				1.4	0.2	5.8	2.3	14.4
30-34		0.0				1.7	0.4	3.8	1.7	8.6
35-39	0.4	0.1	3.4	1.2	9.7	2.3	1.1	2.0	1.0	4.1
40-44	6.1	1.0	6.0	4.4	8.1	4.2	2.3	1.9	1.1	3.2
45-49	33.6	7.8	4.3	3.8	4.9	10.4	4.4	2.4	1.7	3.4
50-54	155.5	47.0	3.3	3.1	3.5	17.1	7.4	2.3	1.7	3.1
55-59	476.0	165.5	2.9	2.8	3.0	25.2	11.9	2.1	1.6	2.7
60-64	1019.3	413.5	2.5	2.4	2.5	32.6	16.0	2.0	1.6	2.6
65-69	1626.7	753.6	2.2	2.1	2.2	32.7	21.0	1.6	1.2	2.1
70-74	1748.3	918.8	1.9	1.8	2.0	31.1	26.1	1.2	0.8	1.8
75-79	1577.9	945.0	1.7	1.6	1.8	61.4	26.2	2.3	1.5	3.7
80-84	1556.5	955.5	1.6	1.4	1.9	63.0	29.8	2.1	0.8	5.7

RR: Rate ratio; CI: confidence interval.

Supplementary Table 5. Age-specific incidence rate and rate ratio of bladder cancer and melanoma in population with and without family history of concordant cancer

	Bladder					Melanoma				
	Incidence rate per 100 000 person years		Rate ratio (Familial/Non-familial)			Incidence rate per 100 000 person years		Rate ratio (Familial/Non-familial)		
Age at diagnosis (years)	Familial	Non-familial	RR	95% CI		Familial	Non-familial	RR	95% CI	
20-24	0.4	0.3	1.4	0.5	4.5	8.0	3.2	2.5	1.9	3.2
25-29	1.0	0.5	2.3	1.1	4.6	18.8	5.7	3.3	2.8	3.9
30-34	1.7	0.7	2.4	1.4	4.2	22.8	9.4	2.4	2.1	2.8
35-39	2.2	1.4	1.5	0.9	2.5	34.8	13.7	2.5	2.2	2.9
40-44	5.4	3.0	1.8	1.3	2.6	50.5	18.9	2.7	2.4	3.0
45-49	10.5	5.5	1.9	1.5	2.4	57.9	24.1	2.4	2.1	2.7
50-54	21.4	10.7	2.0	1.7	2.4	69.2	28.5	2.4	2.2	2.7
55-59	37.8	20.3	1.9	1.6	2.2	84.5	35.7	2.4	2.1	2.7
60-64	70.7	35.3	2.0	1.8	2.3	120.4	44.5	2.7	2.4	3.0
65-69	97.3	55.4	1.8	1.5	2.0	160.9	57.5	2.8	2.5	3.2
70-74	148.0	82.9	1.8	1.6	2.1	159.6	69.3	2.3	2.0	2.7
75-79	177.8	109.1	1.6	1.3	2.0	223.2	89.2	2.5	2.0	3.1
80-84	235.3	135.8	1.7	1.2	2.5	242.1	118.8	2.0	1.2	3.4

RR: Rate ratio; CI: confidence interval.
